# Occupational exposure to blood and body fluids and associated factors among health care workers at the University of Gondar Hospital, Northwest Ethiopia

**DOI:** 10.1186/s12199-019-0769-9

**Published:** 2019-03-09

**Authors:** Jemal Yasin, Roman Fisseha, Feleke Mekonnen, Ketsela Yirdaw

**Affiliations:** 1 Department of Nutrition, Institute of Public Health, College of Medicine and Health Sciences, University of Gondar, P.O. Box 196, Gondar, Ethiopia; 2 Department of Microbiology, School of Biomedical and Laboratory Sciences, College of Medicine and Health Sciences, University of Gondar, P.O. Box 196, Gondar, Ethiopia; 3 Department of Clinical Chemistry, School of Biomedical and Laboratory Sciences, College of Medicine and Health Sciences, University of Gondar, P.O. Box 196, Gondar, Ethiopia

**Keywords:** Occupational exposure, Health care workers, Blood and body fluids

## Abstract

**Background:**

Occupational exposure to blood and body fluids (BBFs) is a serious concern for health care workers (HCWs) and presents a major risk factor for transmission of infectious diseases such as hepatitis B virus (HBV), hepatitis C virus, and human immune deficiency virus. The main objective of this study was to assess the magnitudes of occupational exposure of blood and body fluids and associated factors among health workers at the University of Gondar Hospital.

**Methods:**

An institution-based cross-sectional study was conducted from 1 February to 31 May 2017 at the University of Gondar Hospital. A total of 282 health care workers were selected by simple random sampling technique. Descriptive data was presented as absolute number with percentage, and multivariate analysis was used to assess the statistical association between associated factors and occupational exposure to BBFs. A *P* value of < 0.05 was considered as statistical significant.

**Result:**

A total of 282 HCWs participated with the mean (±SD) age of 30.51 ± 5.86 year. Of the total, 58.5% (165) and 42.2% (119) of the study participants had been exposed to BBFs splash and needlestick injury (NSI) in their lifetime, respectively. However, 39.0% (110) and 20.6% (58) of the HCWs were exposed to splash and NSI in the past 1 year, respectively. Not wearing eye goggle, lack of training on infection prevention, taking of HBV vaccination, and recapping of used needle were risk factors associated with BBFs splash exposure, whereas taking HBV vaccination and recapping of used needle were associated risk factors with NSI exposure.

**Conclusion:**

This study showed a high percentage of occupational exposure to blood and body fluids among health care workers. Not wearing eye goggle, HBV vaccine status, and recapping needles were found to be independent predictors of occupational exposure to BBFs among HCWs. Therefore, relevant stakeholders need to formulate strategies to create a favorable working environment and increase their adherence to universal precautions.

## Background

Occupational exposure to blood and body fluid is the accidental contact with blood and body fluids during a medical intervention by HCWs. These unintended exposures to BBFs carry the risk of infection by various blood-borne viruses. It constitutes a major risk for the transmission of infections such as human immune deficiency virus (HIV), HBV, and hepatitis C virus (HCV). This is one of the serious public health problems that HCWs encounter [1].

HCWs are at high risk of being infected with various diseases transmitted by blood and body fluids due to frequent exposure to biological materials and patient’s body fluids. Needle injuries and injuries due to cutting, biting, or splashing incidents are some of the ways HCWs encounter during their daily activities [2]. The frequency of needlestick injuries and high prevalence of blood-borne diseases in the general population have a great impact on the exposure of different infection agent risk among HCWs [3]. Infectious complications associated with needle stick injury can result in a variety of serious and stressing consequences ranging from mild to extreme anxiety among HCWs [4]. It is very important that HCWs undergo follow-up assessments after being exposed to BBFs for the detection and early treatment of acute infection, such as HCV [5].

Blood of patient with HBV contains the highest HBV level than other body fluids and is the most important source of transmission in the health care center. Cerebrospinal fluid, synovial fluid, pleural fluid, peritoneal fluid, pericardial fluid, and amniotic fluid potential are considered also potentially infectious [6]. Currently, HBV is the only one that has a vaccine from three serious viral infections (HBCV, HCV, and HIV) [7]. HCWs who have received hepatitis B vaccine are at almost no risk for infection [8].

The World Health Organization estimated that 3 million are exposed to blood-borne viruses each year and 90% of the exposures occur in the developing countries [9]. In developing countries, HCWs are at serious risk of infection from blood-borne pathogens particularly HBV, HCV, and HIV because of the high prevalence of such pathogens in general population, particularly sub-Saharan Africa [10].

Occupational hazards faced by HCWs in Ethiopia have received increasing attention but existing surveillance system and HCWs responsiveness for safety precautions are insufficient to describe the scope and extent of occupational exposure to the infectious agent that HCWs experience, the outcomes of these exposures and injuries, and the impact of preventive measures. So the aim of our study was to show the magnitude of occupational exposure to BBFs and to point out the main associated factors. Hence, these findings would provide pertinent information to reduce the exposure of HCWs to blood and body fluids. Moreover, our study provides current updated and baseline information, as well as recommendations for further corrective actions by researchers, governmental, and non-government responsible bodies and other stack holders.

## Methods

The study was conducted in Gondar town, Northwest Ethiopia, which is located 737 km from Addis Ababa the capital city of Ethiopia. The University of Gondar Hospital is a referral hospital which gives medical service to more than 5 million inhabitants in and around Gondar. Currently, it has 446 functional beds for admitting patients and it has senior level to medium level professionals working at pediatrics, surgery, gynecology, psychiatry, HIV care, laboratory, and other service delivery centers.

A cross-sectional study design was conducted to assess occupational exposure on blood and body fluids on HCWs at the University of Gondar Teaching Referral Hospital. The study was conducted from 1 February 1 to 31 May 2017. Single population proportion formula was used by using 62.9% of the prevalence of occupational exposure to BBFs in the previous study [4]. By considering a 5% margin of error and 95% confidence interval, a total of 282 health care workers were obtained. A stratified sampling technique was used to distribute total sample size based on their profession. The study participants were selected by a simple random sampling technique.

A self-administered structured questionnaire was used to collect information about socio-demographic characteristics, HBV vaccination standard precautions use, working environment, and occupational exposure of HCW to BBFs based on the previous studies [11, 12]. HCWs were considered as exposed to NSI if the HCW had a history of one or more of a needlestick or sharps injury whereas HCWs are considered exposed to BBFs splash if the HCW had a history of a splash of any body fluids onto their mucous membranes or skin. In this study, lifetime exposure indicates exposure of the HCWs to BBFs in their career whereas the past one was to indicate recent exposure to BBFs. All the study participants were informed about the purpose of the study and informed consent was obtained from the participants.

Data were entered, cleaned, and analyzed using SPSS for window, version 20 (SPSS Inc., Chicago, IL, USA) statistical package software. Descriptive statistics like frequencies and proportions were used to summarize the data. Crude odds ratio (COR) and adjusted odds ratio (AOR) with their 95% confidence interval were expressed to describe the association of risk factors with BBFs exposure in univariate and multivariate analysis respectively. Univariate analysis was employed to examine the relationship between the dependent variables and independent variables. Those variables with observed association of *P* < 0.25 on univariate analysis were further treated by multivariate analysis in order to adjust for possible confounders. A *P* value < 0.05 was considered significant.

## Results

### Socio-demographic characteristics of health care workers

A total of 282 health care workers participated in this study; a response rate of 96.9%. Among the respondents, 53.9% (152) were males. The age range of study subjects was from 23 to 53 years with the mean (±SD) age of 30.51 ± 5.86 years. Regarding the educational status, 87.2% (246) of HCWs had a bachelor degree and above. From the total HCWs, 45% (127) had experience between 2 and 5 years of service (Table 1).

**Table 1:** socio-demographic characteristics of health care workers

Variables	Result	Frequency (#)	Percentage (%)
Sex	Male	152	53.9
	Female	130	46.1
Age	18–30	169	59.9
	31–40	97	34.4
	> 40	16	5.7
Educational status	Certificate and diploma	36	12.8
	Degree and above	246	87.2
Profession	Nurse	180	63.8
	Diagnostic laboratory	27	9.6
	Medical doctor	13	4.6
	Midwife	27	9.6
	Others	35	12.4
Department	Outpatient	67	23.8
	Injection and dressing	16	5.7
	Surgical ward	18	6.4
	Operation room	27	9.6
	Pediatric ward	17	6.0
	Gynecology ward	46	16.3
	Medical ward	30	10.6
	Diagnostic laboratory	25	8.9
	Others	36	12.8
Work experience	< 2 years	57	20.2
	2–5 Years	127	45.0
	6–9 years	72	25.5
	≥ 10 years	26	9.2

### Prevalence of occupational exposure to BBFs

Of the total, 58.5% (165) study participants had been exposed to BBFs splash in their lifetime. However, 39.0% (110) of HCWs were exposed to BBFs splash in the past year. Histories of needlestick injury over their lifetime and in the past year were 42.2% and 20.6%, respectively (Table 2).

**Table 2:** Frequency of occupational BBFs splash and NSI exposure among health care workers

Variables	Result	Frequency (#)	Percentage (%)
Lifetime occupational exposure to splash	Yes	165	58.5
	No	117	41.5
The past 1-year occupational exposure to splash	Yes	110	39.0
	No	172	61.0
Lifetime occupational exposure to NSI	Yes	119	42.2
	No	163	57.8
The past 1-year occupational exposure to NSI	Yes	58	20.6
	No	224	79.4

*BBFs* blood and body fluids, *NSI* needlestick injury

### Distribution of common factors with occupational exposures of blood and body fluids

From the study participants, 39.36% (111) have been trained in occupational infection prevention. Seventy-seven percent (65) of the study participants responded that there were not enough personal protective equipment (PPE) available over the past year which refers basic wear to create a barrier between personnel and germs such as *wearing gloves*, *masks*, *eye protection*, and *clothing.* The presence of safety signs in the working area was answered by 25.9% (73) of the study participants. The majority (94%) of the study participants had used gloves during the last health care procedure. Seventy-two percent (203) of the study participants had enough hand washing facilities in their working area. Nearly 90% (252) of HCWs washed their hands before and after any health care procedure as well as handling and processing of BBFs. A total of 55.3% (156) study participants were vaccinated for HBV. 70.6% (199) of HCWs did not have information on the availability of an infection prevention committee in the health institution. Moreover, 173 study participants responded that the workplace was not safe for the prevention of occupational exposure to BBFs (Table 3).

**Table 3:** Distribution of common factors to occupational exposures of blood and body fluids among health care workers

Variables	Results	Frequency (#)	Percentage (%)
Training on occupational infection prevention	Yes	111	39.36
	No	171	60.64
Practicing universal precaution and safety	Yes	124	44.0
	No	158	56.0
Availability of adequate PPEs	Yes	65	23.0
	No	217	77.0
Availability of safety signs in the workplace	Yes	73	25.9
	No	209	74.1
Wearing of gloves during handling and processing of BBFs	Yes	265	94.0
	No	17	6.0
Wearing of eye goggle during handling and processing of BBFs	Yes	43	15.2
	No	239	84.8
Safety of the workplace in the prevention of exposure to BBFs	Yes	109	38.7
	No	173	61.3
Availability of adequate hand washing facilities in the workplace	Yes	203	72.0
	No	79	28.0
Washing of hands before and after any procedure or process	Yes	252	89.4
	No	30	10.6
Availability of an infection prevention team	Yes	83	29.4
	No	199	70.6
HBV vaccinated	Yes	156	55.3
	No	126	44.7
Recapping of the used needle	Yes	126	44.7
	No	156	55.3
Applying of universal safety precaution standards	Yes	83	29.4
	No	199	70.6

*BBFs* blood and body fluids, *HBV* hepatitis B virus, *PPE* personal protective equipment

### Factors associated with occupational exposure

The univariate analysis showed significant association between BBFs splash exposure and the following risk factor’s age, department, training on infection prevention, wearing of eye goggle, and availability of enough washing facilities, the presence of safety sign, existence of infection prevention committee, having HBV vaccination, and recapping of used needles a value of *P* < 0.25 (Table 4). However, age, department, training on infection prevention, wearing of eye goggle, the presence of safety sign and committee, having of HBV vaccination, and recapping of the used needle were significantly associated with NSI exposure (Table 5).

**Table 4:** Multivariate logistic regression analysis of risk factors associated with blood and body fluids in the past year exposure to blood and body fluids

Variables	Exposure to BBFs		COR (95% CI)	AOR (95% CI)	*P* value
	Yes	No			
Age (in years)					
18–30	93	76	1.00		
31–40	58	39	1.22 (0.73–2.02)		
> 40	14	2	5.72 (1.26–2595)		
Job					
Nurse	99	81	1.00		
Laboratory	20	7	2.34 (0.94–5.8)		
Doctor	11	2	4.5 (0.97–20.89)		
Midwife	15	12	1.02 (0.45–2.31)		
Others	20	15	1.09 (0.53–2.270)		
Department					
Outpatients	33	34	1.00		
Injection and dressing	12	4	3.09 (0.91–10.56)		
Surgical ward	12	6	2.06 (0.69–6.13)		
Operating theater	13	14	0.96 (0.39–2.34)		
Pediatrics	9	8	1.16 (0.4–3.34)		
Gynecology	30	16	1.93 (0.89–4.19)		
Laboratory	18	7	2.56 (0.98–7.17)		
Medical ward	21	9	2.4 (0.96–6.01)		
Others	17	19	0.92 (0.41–2.07)		
Experience (in years)					
< 2	28	28	1.00		
2–5	75	52	1.44 (0.77–2.71)		
6–9	42	30	1.4 (0.69–2.83)		
> = 10	20	6	3.33 (1.16–9.55)		
Training on infection prevention					
Yes	79	32	1.00	1.00	0.006
No	86	85	0.41 (0.25–0.68)	0.47 (0.27–0.8)	
Practicing universal precaution and safety					
Yes	80	44	1.00		
No	85	73	0.64 (0.4–1.04)		
Wearing of eye goggle					
Yes	20	23	1.00	1.00	0.02
No	145	94	1.77 (0.92–3.41)	2.29 (1.14–4.6)	
Availability of enough hand washing facilities					
Yes	125	78	1.00		
No	40	39	0.64 (0.38–1.08)		
Presence of safety sign					
Yes	36	37	1.00		
No	129	80	1.66 (0.97–2.84)		
HBV vaccination					
Yes	104	52	1.00	1.00	0.025
No	61	65	0.47 (0.29–0.76)	0.55 (0.33–0.93)	
Recapping of used needle					
Yes	91	37	2.66 (1.62–4.37)	2.22 (1.32–3.74)	0.003
No	74	80	1.00	1.00

**Table 5:** Multivariate logistic regression analysis of risk factors associated with NSI in the past year exposure to blood and body fluids

Variables	Exposure to BBFs		COR (95%CI)	AOR (95%CI)	*P* value
	Yes	No			
Age (in years)					
18–30	64	105	1.00		
31–40	44	53	1.36 (0.82–2.26)		
> 40	11	5	3.61 (1.2–10.86)		
Department					
Outpatients	26	41	1.00		
Injection and dressing	11	5	3.47 (1.08–11.13)		
Surgical ward	9	9	1.58 (0.55–4.49)		
Operating theater	11	16	1.08 (0.44–2.7)		
Pediatrics	7	10	1.1 (0.37–3.26)		
Gynecology	24	22	1.72 (0.81–3.68)		
Laboratory	7	18	0.61 (0.23–1.67)		
Medical ward	10	20	0.79 (0.32–1.95)		
Others	14	22	1 (0.44–2.3)		
Training on infection prevention					
Yes	55	56	1.00		
No	64	107	0.61 (0.38–0.99)		
Wearing of eye goggle					
Yes	14	29	1.00		
No	105	134	1.62 (0.82–3.23)		
Availability of enough hand washing facilities					
Yes	90	113	1.00		
No	29	50	0.73 (0.43–1.24)		
Presence of safety sign					
Yes	25	48	1.00		
No	94	115	1.57 (0.9–2.73)		
Presence of infection prevention committee					
Yes	40	43	1.00		
No	79	120	0.71 (0.42–1.19)		
HBV vaccination					
Yes	80	76	1.00	1.00	0.006
No	39	87	0.43 (0.26–0.7)	0.49 (0.3–0.82)	
Recapping of used needle					
Yes	71	57	2.75 (1.69–4.48)	2.45 (1.49–4.03)	0.000
No	48	106	1.00		

In the multivariate analysis, training on infection prevention (AOR = 2.17, 95% CI 1.25, 3.7), lack of wearing eye goggle (AOR = 2.29, 95% CI 1.14, 4.6), having HBV vaccination (AOR = 1.82, 95% CI 1.08, 3.03), and recapping of used needle (AOR = 2.22, 95% CI 1.32, 3.74) were found to be risk factors associated with occupational exposure to splash (Table 4), whereas only having HBV vaccination (AOR = 2.04, 95% CI 1.22, 3.33) and recapping of used needle (AOR = 2.45, 95% CI 1.49, 4.03) were found to be risk factors associated with NSI exposure (Table 5).

## Discussion

The exposure of HCWs to BBFs during intervention has exposed them to different blood-borne diseases which in turn have had an impact on their health and health care services in many countries, particularly developing countries with limited human resources and poor infrastructure [13]. HCWs at the University of Gondar Hospital have to deal with a high load of patients; this fact combined with the urgency of some interventions contributes to this high prevalence of BBFs among studied groups.

In this study, 58.5% of HCWs were exposed to BBFs splash in their lifetime, which was lower than findings from Serbia (66%), Iran (74%), India (73%), and Bahir Dar (74%) [12, 14–16]. However, this study indicates higher percentage of HCWs exposed to blood and body fluids compared with the studies conducted in Kenya and Eastern Ethiopia [11, 17]. The reasons for this variation could be due to lack of regular training about safety precaution sand infection prevention, inadequate supervision by health administrators, and infrastructure development.

In the present study, 39.0% (110) of HCWs were exposed to BBFs splash in the past 1 year which was lower than the study conducted in Bahir Dar town (65.9%) and previous study in Gondar University Hospital (62.9%) [4, 12]. On the contrary, our findings revealed a higher percentage of HCWs exposed to BBFs when compared to studies done in Kenya (25%), Brazil (7%), and Bale Zone (19.1%) [17–19]. This difference could possibly be due to the absence of continuous training on prevention of occupational infection, difference in infrastructure development, and low functionality of existing infection prevention committee as well as the experience of HCWs to adhere to standard occupational safety precautions.

National Institute of Occupational Safety and Health in the USA identifies the following as predisposing factors to needlestick injuries: over-use of injections, unnecessary sharps, lack of PPE supplies, failure to use sharps container immediately after use, poorly trained staff, needle recapping, no engineering control, such as safe needle devices, passing instruments from hand to hand in the operating room, and lack of hazard awareness [20]. This was in agreement with our findings in which 67.4% of staffs are untrained, facilities lack safety devices, and needle recapping is still practiced.

Our finding showed that 42.2% (119) of study participants were injured by needlestick injury in their lifetime, which was lower than studies done in India (63%) and Bahir Dar town (50%) [12, 16]. However, our finding on the needlestick injury in the past 1 year, 20.6% (58) was almost comparable with finding from Bale Zone (19%) [19]. On the other hand, our study was higher than studies from Iran (8%) and Dire Dawa [11, 15]. This difference might be due to variations in the health care setting, the availability of PPE in health care facilities, and training about infection prevention.

In this study, 44% (124) of the HCWs practiced universal precaution and safety, which was slightly higher than the study conducted in public health facilities in Mekelle special zone [21]. On the other hand, 39.36% (111) of study participants had training on infection prevention, and the finding is similar to the study conducted in India [22]. However, the level of training on infection prevention was lower compared to studies in Eastern Ethiopia and Debrebirhan Town [11, 23]. This could be due to the lack of commitment, limited budget available to support training, and capacity building.

Hepatitis B vaccination coverage among HCWs in our study was 55.3% (156), which was higher than in a study conducted in a provincial hospital in Kenya, where only 40% of HCWs was vaccinated [17]. Though there was a slightly higher coverage of HBV vaccination, still our findings showed below WHO expectations, 100%. The potential reasons for the low HBV vaccine coverage might be the unavailability of the vaccine at the health facility due to high cost and irregular distribution, especially in the developing countries.

Even though the availability of HBV vaccine is a good progress, HCWs have to protect themselves from other dangerous pathogens like HIV and HCV due to exposure of HCWs to BBFs. In our study, HCWs being vaccinated were 1.82 times (AOR = 1.82, 95% CI 1.08, 3.03) and 2.04 times (AOR = 2.04, 95% CI: 1.22, 3.33) more exposed to splash and NSI than their counterparts, respectively. The reasons might be negligence and being careless during blood and body fluid samples process. However, HCWs have to develop awareness that exposure to splash and NSI brings a wide range of blood-borne dangerous pathogens such as HIV and HCV.

The previous study in occupational exposure and behavior of health care workers in Ethiopia shows needle recapping as a major cause of NSI [11]. Our study showed a higher prevalence of needle recapping after use (44.7% (126)), which was greater than the studies conducted in Nigeria (35.3%) [24] and Northern Ethiopia (34.7%) [25]. HCWs who practiced needle recapping were 2.45 times more likely to experience an injury than who did not recap needles after use (AOR = 2.45, 95% CI 1.49, 4.03). The reason for this difference may be related to improper practice and lack of adequate training on infection prevention, negligence, workload, and lack of safety devices.

Showing the current picture of occupational exposure to BBFs could be taken as the strength of the study. However, this study has limitations due to a cross-sectional study design in which social desirability bias is a problem, and also this study was based on self-report about previous 1 year and lifetime occupational exposure to BBFs; this may affect the result by recall bias.

## Conclusion

This study showed higher percentage of occupational exposure to blood and body fluids among health care workers in the study area. Lack of training on prevention of occupational infection, HBV vaccine status, and recapping needles were found to be independent predictors of occupational exposure to BBFs among HCWs. Based on the current assessment, relevant stakeholders need to provide training on prevention of occupational infection to HCWs, arrange provision of infection prevention supplies, formulate strategies to create a favorable working environment, and increase their adherence to universal precautions.

## Abbreviations

HBV: Hepatitis B virus
HCV: Hepatitis C virus
HCWs: Health care workers
HIV: Human immune deficiency virus
NSI: Needlestick injury
PPE: Personal protective equipment
